# Older adults’ perceptions of contexts surrounding their social participation in a digitalized society—an exploration in rural communities in Northern Sweden

**DOI:** 10.1007/s10433-020-00558-7

**Published:** 2020-02-11

**Authors:** Caroline Fischl, Nina Lindelöf, Helena Lindgren, Ingeborg Nilsson

**Affiliations:** 1 Department of Community Medicine and Rehabilitation, Occupational Therapy, Umeå University, 901 87 Umeå, Sweden; 2 Department of Rehabilitation, School of Health and Welfare, Jönköping University, Box 1026, 551 11 Jönköping, Sweden; 3 Department of Community Medicine and Rehabilitation, Physiotherapy, Umeå University, Umeå, Sweden; 4 Department of Computing Science, Umeå University, Umeå, Sweden

**Keywords:** Computer, Digital technology, Information and communication technology, Internet, Older people, Social activities

## Abstract

Social participation and digital engagement can contribute to health and well-being among older adults. Because of older adults’ decline in abilities, coupled with complex technology and its perceived insufficient relevance to daily life, there is a need to create and tailor social opportunities and services that are supported by digital technologies for older adults to continue participating in society. Thus, it becomes relevant to explore older adults’ perceptions about contexts surrounding their social participation in a digital society. This exploration used a qualitative research design with focus group interviews and qualitative content analysis. Eighteen older adults, aged 66–81 years, from rural communities in Northern Sweden, participated in this study. The analysis resulted in three categories: *experiencing conditions for social participation in a state of flux, perceiving drawbacks of urbanization on social participation,* and *welcoming digital technology that facilitates daily and community living.* These categories were encapsulated in the theme—*the juxtaposition of narrowing offline social networks and expanding digital opportunities for social participation.* The findings suggested that co-creating usable digitalized services and facilitating satisfactory use of digital technologies could support older adults’ social participation through activities that they find relevant in their lives, and subsequently, might enable them to live longer at home.

## Background

Social participation is concerned with involvement in activities that provide interactions with others, a community, or society at large (Levasseur et al. 2010). In a society that is rapidly becoming digitalized (Davidsson and Thoresson 2017; Regeringskansliet 2017), social participation occurs increasingly more often with and through the use of digital technologies. Digital technologies, according to Fischl et al. (2017), include personal computers, smart telephones, and computer tablets, the software in these devices, as well as the Internet and World Wide Web. As digitalization affects all life areas and sectors in society (Digitaliseringskommissionen 2016), digital technologies can enable people to be connected to and participate in society according to their preferences (Larsson et al. 2013; Leist 2013; Olphert and Damodaran 2013). Both social participation and digital technology have been recognized as having potential to promote health and well-being among older adults (World Health Assembly 2018; World Health Organization 2015).

However, this potential can be thwarted by digital inequalities that result from varying access to and use of digital technologies. In Sweden where access to the Internet at home has sturdily increased over the years, digital inequalities are more likely to occur between daily users and rare or non-users of the Internet (Davidsson et al. 2018). The majority of rare and non-users of the Internet are adults over 65 years (Internetstiftelsen 2019). Among these older adults are the *digitally disengaged* who have reduced or completely discontinued use of digital technologies due to decreased capacity, increasing complexity of technology, and inadequate relevance of technology in their lives (Olphert and Damodaran 2013; Selwyn 2006). Digital disengagement makes it even more difficult for older adults to keep up with technological advancements, further narrowing their opportunities.

Ragnedda (2017) argued that digital inequalities can be exacerbated by existing social inequalities, such that individuals who are more advantaged in society have more possibilities of benefitting from digitalization compared to individuals who are disadvantaged. Persons who participate in more leisure activities are more likely to use the Internet (Näsi et al. 2012). In contrast, older adults with limited social contacts have less opportunities to use digital technology for social engagement (Ihm and Hsieh 2015). Furthermore, Davidsson et al. (2018) reported that those who do not or rarely use the Internet in Sweden more often live in rural communities, have lower educational attainment, have lower income, and are female. In turn, digital inequalities tend to aggravate existing social inequalities (Ragnedda 2017). Varying access to and use of digital technologies affect which benefits individuals derive from being connected and participating in society. To exemplify, people who seldom or never use the Internet have reported feelings of reduced participation in society (Davidsson et al. 2018). Ihm and Hsieh (2015) suggested that understanding digital inequalities among older adults requires “a more nuanced approach” (p. 1134) and a closer look at older adults’ specific contexts. This paper adopts the World Health Organization (2002) definition of *context*, which refers to internal personal and external environmental factors.

Creating and tailoring social participation opportunities in a digital society can be better understood by gaining insights about older adults’ contexts. Therefore, the objective of this study was to explore older adults’ perceptions about contexts surrounding their social participation in a digital society.

## Methods

The study’s qualitative research design used focus group methodology for data collection and qualitative content analysis for data analysis. The researchers’ backgrounds in various disciplines—computer science, gerontology, human–computer interaction, occupational therapy, and physiotherapy—in addition to NL and IN’s experiences in using qualitative methodology provided a broader perspective in data collection and analysis.

### Participants

Invitation to participate in this study was in the form of a paid advertisement in a local bulletin with a free weekly distribution to its residents, a notice posted in bulletin boards in community gathering spaces, and oral presentations in community and hobby organizations whose memberships comprised mainly older adults. It was extended to older adults who were 65 years or older, curious about the Internet, and interested to develop Internet-based services for older persons. It was clarified in the invitation that neither access to nor experience in digital technologies was required. The invitations were distributed in rural communities within two municipalities in Northern Sweden with existing broadband infrastructure.

Twenty-one older adults initially responded positively to the invitation. Among them, 16 persons indicated their availability during the data collection periods and were selected through purposive sampling. Two additional persons were recruited through snowball sampling. An exclusion criterion was marked impairment in receptive and expressive language, although no respondent to the invitation was excluded due to this criterion. In total, seven men and 11 women, aged 66–81 years (median = 71.5 years), participated. They had diverse educational and work backgrounds (Table 1), based on responses on a questionnaire collecting information on demographics and digital technology access and use. Information about health was not included in the questionnaire, although it was observed that all participants were able to walk independently inside the interview venues and communicate coherent ideas. They were assigned to one of four focus groups, each consisting of four to six participants. The focus groups were formed based on the communities where the participants lived and their availability for the interviews.

**Table 1:** Background information about the participants

Background information	Men *n* = 7	Women *n* = 11
Living situation		
Living alone	2	3
Living with spouse, partner, or child	5	8
Number of children		
1	1	0
2	4	9
3	2	2
Number of children living in the same municipality but not in the same house		
0	3	2
1	2	5
2	2	4
Residence		
Single unit detached housing, owned	5	8
Single unit detached housing, rented	1	1
Multi-unit housing, rented	0	2
No response	1	0
Highest level of education attained^a^		
Primary education	1	5
Lower secondary education	0	4
Upper secondary education	3	0
Post-secondary non-tertiary education	2	0
Tertiary education—level unspecified	1	0
No response	0	2
Occupational field before retirement^b^		
Occupations requiring advanced level of higher education	3	1
Occupations requiring higher education qualifications or equivalent	1	1
Administration and customer service clerks	0	3
Service, care and shop sales workers	0	3
Agricultural, horticultural, forestry, and fishery workers	1	0
Building and manufacturing workers	2	0
Mechanical manufacturing and transport workers, etc.	0	1
Elementary occupations	0	1
Unspecified working tasks (self-employed)	0	1

^a^Classification based on the International Standard Classification of Education 2011 (United Nations Educational, Scientific, and Cultural Organization 2012)

^b^Classification based on the Swedish Standard Classification of Occupations 2012 (Statistics Sweden 2012)

All participants had access to at least one of the following devices at home: stationary computer, laptop computer, or tablet/handheld computer. Two women had access to two types of computer, while one man had access to all three types. Computer use ranged from daily to occasional use. Four participants indicated smartphone use (Table 2).

**Table 2:** Summary of access to and use of digital technologies among the participants

	Has access^a^ to technology at home		Uses technology daily		Uses technology occasionally	
	Men	Women	Men	Women	Men	Women
Stationary computer	5	5	4	1	1	4
Laptop computer	2	5	1	2	1	3
Tablet/handheld computer	2	3	1	2	1	1
Printer	1	4	1	0	0	6
Mobile telephone	4	8	4	5	0	2
Smart telephone	1	3	2	3	0	0
Game console	0	0	0	0	0	0
Internet	4	7	4	4	0	3

^a^Note that some numbers may not correspond between access and use, as information about access to technology outside of one’s home was not gathered, and participants might not have used technology even if they have access to it

### Data collection

Focus group methodology was chosen in order to stimulate discussion and elaboration of ideas between the participants and to gain insight into how participants talk about ideas (Dahlin-Ivanoff and Hultberg 2006; Doody et al. 2013a; Stewart et al. 2007). Recommendations (Dahlin-Ivanoff and Hultberg 2006; Doody et al. 2013a, c; Skjutar et al. 2010) were considered in planning the group formation and interview structure. Two semi-structured interviews for each focus group, 1 to 4 weeks apart, were held. The main questions in the interviews were about daily activities, social networks and activities, participation in society, digital technology, and existing and desired services in the community. The latter two topics were discussed more in succeeding interviews. In all interviews, CF served as moderator and facilitated discussions. IN and another researcher each served as assistant moderator in two focus groups and were responsible for concluding each interview with a summary of what had been discussed. The summary gave participants an opportunity to reflect on the discussion and to expound on the ideas raised. Each interview lasted 1.5 to 2 h, including a coffee break.

All interviews were conducted in local community centers during January 2014–June 2016. Time intervals between interviews enabled the researchers to reflect on the ideas that emerged from each interview and plan discussion topics in subsequent interviews to achieve data saturation, as recommended by Doody et al. (2013b). The interviews were audio-recorded and transcribed verbatim.

### Data analysis

Qualitative content analysis (Graneheim and Lundman 2004) and an inductive approach to analyze the manifest content in the interviews (Graneheim et al. 2017) were used. First, the recordings and transcripts were reviewed to gain an understanding of each discussion as a whole. In the transcripts, CF wrote notes and highlighted phrases that provided insight about ideas that participants agreed or disagreed with or had in common. Each transcript was broken down into meaning units and coded, from which subcategories were formulated. CF, NL, and IN coded and formulated subcategories for various transcripts separately and then discussed together their initial analyses. In iterative dialogs, all authors were involved in abstracting and interpreting, while moving back and forth between raw data, subcategories, categories, and the theme. The analysis also involved phrasing findings in a way that is comprehensible to and usable by a multidisciplinary team (Sandelowski and Leeman 2012).

## Findings

The findings can be encapsulated in an overall theme—*The juxtaposition of narrowing offline social networks and expanding digital opportunities for social participation.* Within the discussions, participants revealed reduced opportunities for social interaction, which they ascribed to prioritizing home activities, dwindling social groups, and increasing computerized access to services. Concomitantly, participants acknowledged increasing possibilities to use digital technologies in daily life. Three categories, each based on subcategories, provided the frame for the theme and represented contextual factors (Table 3).

**Table 3:** Theme, categories, and subcategories formed

Theme	The juxtaposition of narrowing offline social networks and expanding digital opportunities for social participation		
Categories	Experiencing conditions for social participation in a state of flux	Perceiving drawbacks of urbanization on social participation	Welcoming digital technology that facilitates daily and community living
Subcategories	Prioritizing time for doing preferred activities Changing social and activity spaces and expanding online Adapting social groups and codes influencing engagement Faltering participation despite membership in community or organization Wanting to contribute to society	Receding contact with service providers creating insecurity Drawing key social players away through technology and urbanism Viewing driving as freedom	Finding relevance in available information Preferring technology based on need for personal contact and feelings of security Desiring service and technology developments as societal support Weighing interests against usability and data protection

### Experiencing conditions for social participation in a state of flux

Participants discussed that activities had taken longer time as they got older and that their days were often occupied with mundane activities at home, like following current events and sports, solving puzzles, and doing household tasks. Some explained that they prioritized home improvement activities, which did not leave much time for socialization. It appeared that doing prioritized activities at one’s own pace was important.

2b W5^1^: I think time is too short. There is so much I would like to do, but I might not really manage…

W4: If one is not pressured, then things can take time. It is so nice not to be stressed.

W5: I can just sit and enjoy half a day deciding on what I want to do today.

Participants discussed how social spaces had changed over time. For instance, they reminisced about having social dances and family gatherings in an old community building and talked about public places converted from old private properties. Participants acknowledged that their social spaces were not limited to physical environments but included virtual milieus. They compared the telephone, e-mail, videoconferencing tools, and social networking sites for keeping contact with others, specifically family members living far.

1a M1: I had a grandchild in the United States in…

W1: …[city1]! Yes, we had Skype then. We still use it now, even when he doesn’t live in the U.S. anymore…

M2: I have a daughter in [city2], and I have seen her entire house via Skype.

Participants recounted activities with and for family and friends. Some divulged that they discontinued shared activities, like couple dancing, after their mates’ health had declined, while some had decided to regularly meet as long as they were in good health. Nevertheless, participants concurred that they had satisfying social networks, regardless of the size. Participants also discussed how social conventions and habits had affected their opportunities for interaction. For instance, spontaneous visits had been replaced by explicit invitations to others’ homes. They reasoned that occupying one’s time with technology, like television, had taken out the spontaneity in social meetings and that one had to find an excuse for initiating conversations with others. Moreover, participants argued about whether everyday clothes could hinder them from engaging in social meetings.

3a W7: I told my husband, you can go over and talk to the neighbor because he is waving at us. My husband wouldn’t dare to go there because he had garden boots on.

M6: So typical! (laughing).

W8: But it shouldn’t matter.

Participants took turns describing activities in community, interest, and hobby organizations whose members were spread out in the region. They alluded to a decline in organized activities because of organizations’ limited financial resources. Some disclosed having missed out on activities in organizations either unwillingly or willingly. They reasoned that their age, health condition, or difficulty to learn new things contributed to a decline in their participation. Speculations about how old age and health deterioration had caused acquaintances to discontinue their membership were raised. They also remarked that activities were often attended by the same persons, narrowing the likelihood to meet new people. Some participants discussed engagement in an organization as a burden.

1b M1: It seems like organizational activities are dying out…

W2: That’s because nobody wants to get involved.

M1: No one wants to accept the chairmanship…

W2: And nobody wants to be involved in the board.

M1: Before, it was an honorable assignment to be on the board. Nowadays, it is the opposite.

W1: They don’t even come to the meetings because they are afraid they must do something.

Nevertheless, participants discussed that they had a desire to remain independent of others and to continue contributing to society. They conversed about spending time to do charity work and assisting older persons who neither were able to do things nor went out of their homes. Helping others felt satisfying and fun and provided social interaction for themselves and the people they encounter. They also acknowledged that social interaction was important in old age and discussed their concerns about peers who choose not to go out and risk becoming lonely.

3a W6: I go there every day and bring the mail of those who have difficulty to walk. We talk for a while. They think it is fun because they just sit in their flats. Since I can still walk and have a car, I can bring them wherever they want. I exert effort, but at the same time I think it is fun.

W7: I always thought that you do a fantastic job, helping there.

### Perceiving drawbacks of urbanization on social participation

Participants discussed how changes in services affected their social participation. For instance, relocation of the postal office outside the community made it difficult to post Christmas cards. Participants agreed that they needed direct contact with service providers to feel secure and that concentrating services, particularly health care, in urban localities had made them feel less secure about growing old in their communities. Participants discussed how interim doctors replaced retired local doctors and misspent time on repetitive patient history-taking and computer work. It was perceived that one had to be well enough to speak out for oneself or have family to advocate one’s health needs. Furthermore, they agreed the automated telephone access to healthcare services was complicated.

1a W1: I was the one who had to make the phone calls and fix everything for [my mother], even though she could manage herself.

W2: Those who have had a stroke or something similar would have difficulties following instructions.

M2: By the time you pressed [telephone buttons] to where you should be, then the telephone hours are over.

W1: It can happen that you ring right on time, but all time slots are filled.

Participants also discussed how community developments had affected their opportunities for social interaction. One participant divulged that since the municipality had decreased the number of homes for seniors in their area, she had less possibilities to meet and help peers close to home. Another participant added that it had become boring around the neighborhood, having only young neighbors who are away for work or school in the city during the day. Others discussed that more older people were moving to cities from rural areas. Additionally, they reasoned about younger residents moving south or having other interests, explicitly on computers, which had contributed to the decline of local social activities.

1a M1: [Social events] have slowed down now, there were more before.

W2: There is nothing now. But these events come sporadically…

M2: Yes, but doesn’t this have to do more with the general social development, that all jobs are brought to the big cities?

W2: We have enough youth, but they have other interests nowadays… and their need to meet does not seem to be so strong.

With decreasing services and opportunities for close social interactions where they live, participants acknowledged that a car was necessary to do activities, meet people, join social gatherings and courses, and access health care. A participant added that not having a driving license hindered participation in a course in the city. Some reported city parking was difficult. Others suggested the bus as an alternative, but some insisted that taking the bus was more difficult. The car was therefore viewed as “freedom” (1a W2 and 3a M6). Participants explained that without a car or the ability to drive a car, they foresaw becoming sick or lonely and would consider moving to the city.

1b W2: The car is a must when one lives like this.

M2: It is. You know, you go to [city1] to meet people… I realized this when I was out dancing last Friday in [city2]… I wouldn’t be able to go there if I didn’t have a car. I have to move, in that case to have social activities, closer to where there are more people.

### Welcoming digital technology that facilitates daily and community living

Participants discussed a need to keep informed about events and being able to access information through different media from home. There was an agreement that newspapers and radio covered more current events and advertisements from urban localities, which were irrelevant to them. They acknowledged that the local bulletin board was particularly important to community life, although it was discussed that people could find information about their hometowns and current events anytime through the Internet. They disclosed that they could check social networking sites for updates about people they know but disapproved of silly information that was posted.

3a W8: Many [people] share everything. One can get crazy with that.

W6: It’s just nonsense.

Participants attested to existing online services that they found useful, such as borrowing books from the library Web site, ordering goods for home delivery, paying bills directly on one’s online bank account, and authenticating transactions through electronic citizen identification. Yet, participants still expressed preference for personal and physical contact, particularly with bank staff and healthcare professionals. They disagreed about computers’ usefulness in keeping contact with others, but agreed that they preferred hearing someone talk on the telephone and meeting a real doctor in an online meeting. They added that in order to continue living at home, things like technology should function well. Access to emergency care and communication that worked gave them a feeling of security, while technology that did not function well provided no security at all.

1b M2: The only security is the telephone. If that doesn’t work then it doesn’t matter if I have a computer or not.

W1: A security alarm might not work either.

M2: It is, you know, the security for me to be able to live where I live.

Furthermore, participants discussed that they would like more extensive home delivery services for food, groceries, and pharmaceutical products, as well as online healthcare services accessible from home. They also talked about wanting to access entertainment (e.g., concerts and opera) and social activities (e.g., contacting a resource person in a course) through the Internet. Although they themselves do not require home care, they deliberated about preferring to have nighttime camera surveillance in order to live longer in their homes than to move out and receive care elsewhere. When asked what they would need for participation in the future, participants agreed that it was difficult to imagine their future needs when they were “too well” (2b M4 and M5) and that their needs could only be understood when one could not do things anymore. They also discussed how fast technology developed over their lifetime but disagreed on how much they needed to follow technological developments.

3b W7: I remember when I was a child and somebody said, *Think about it. It would surprise me that on one beautiful day we can see each other when we talk on the telephone.* Nowadays, we can do that. I would never have believed it then.

W6: Yes, it has gone unbelievably fast. Can’t wonder why one doesn’t manage to keep up with it. One managed to learn some things, like TV and this (waving her mobile telephone)… But nowadays it has been so fast, that everything becomes outdated in no time.

M6: One should have so little technology as possible. One would manage anyway.

Participants admitted getting discouraged, discontinuing activities, and turning off the computer when they encountered technology issues, like having a slow device or non-functioning interfaces and getting too many junk e-mails. They agreed that computer instructions that were often in English or technical jargon posed additional difficulties. Furthermore, they discussed that they felt suspicious of or threatened by Web sites or programs requiring personal information, as well as worried over fraudulent Internet activities to swindle older people. To solve technical problems, participants acknowledged that they often relied on their grandchildren, even remotely. Some participants described having joined a course to be able to use a computer, while other participants admitted that they were not interested to use computers.

4b W9: I have actually attended some computer course, but it did not lead to me using [the computer]… I am really not interested in it!

W10: It is that which is important—interest and curiosity.

## Discussion

In the exploration of older adults’ perceptions about contexts surrounding their social participation in a digital society, focus group participants reminisced about past situations, discussed perceptions of their current contexts, and contemplated about their future needs. The findings thus revealed a transaction between different factors—individual priorities and preferences, perceptions of one’s health and ability, social networks and opportunities, services, and digital technologies—and its fluidity in time. Main features in this transaction were the decreasing social opportunities and increasing digital applications, thus resulting in the theme. Reduced social opportunities in rural areas have been previously noted (Vogelsang 2016; Winterton et al. 2014). Decreasing social network size has been seen in other studies (e.g., Barnes et al. 2004; Conway et al. 2013), although a recent study (Schwartz and Litwin 2018) indicated increasing social networks among older adults in European communities. An intentional reconstruction of older persons’ social networks could be considered adaptive, according to Carstensen et al. (2003). Contrarily, when reduced unwillingly, it can result in a health decline (Jivraj et al. 2016). Thus, when the narrowing of social networks is unintentional, a need for support emerges.

With regard to the latter element, expanding digital opportunities for social participation has been reported (e.g., Bergström 2017; Olphert and Damodaran 2013). Digital technologies that fit individual needs could enhance older adults’ social participation. However, there remains a risk that only older adults who are more advantaged in society may experience benefits from digital opportunities (Ragnedda 2017). Additionally, expanding digital opportunities may lead to feelings of reduced social participation for older adults who seldom or never use digital technologies (Davidsson et al. 2018). Thus, it is also important to create essential digitalized services and systems that are usable and relevant to older adults who are digitally disengaged or seldom engaged.

The findings revealed that engagement in social activities varied among older adults, which was consistent with other studies. Based on Levasseur et al.’s (2010) taxonomy of social activities, social activities include doing an activity in preparation for connecting to others; being and interacting with others; and doing an activity with others and for others or society (p. 1246). Similarly, Aw et al. (2017) describe activities done alone, activities done with others, and activities performed for others in a continuum of social participation. In this study, participants opted out of social activities to engage in activities they prioritized, like home improvement activities. These activities, either done with someone they live with or done alone to prepare for socializing with others in one’s home, are considered as participation according to Levasseur et al. (2010). Home improvement activities may also be regarded as a way to achieve a sense of security and stability, which are important aspects of healthy aging (Reichstadt et al. 2007).

Furthermore, smaller social groups and reduced engagement in organizations were revealed. Findings relating to satisfaction over small social groups are consistent with the socioemotional selectivity theory (Carstensen et al. 1999, 2003), suggesting that older adults often perceive insufficient time to pursue their goals. Thus, older adults intentionally select more meaningful relations over less relevant contacts, thereby reducing their social networks yet feeling often satisfied (Carstensen et al. 2003). Actively forming satisfying social relations may include appropriate clothing. According to Twigg (2007), clothing is important in older adults as it is an expression of agency, identity, and belongingness. Soiled clothes could indicate “a social and moral decline that may threaten a person’s capacity to remain part of mainstream society” (p. 295). Likewise, reduced engagement in organizations can be related to participants’ selection of more meaningful activities. While being active in organizations can be viewed as opportunities to meet and help others, it could also be experienced as an obligation (Martinson and Minkler 2006). Refusing responsibility in an organization could indicate a demonstration of agency and choice. However, disengagement from social activities is common among older adults who experience health declines, and prolonged social disengagement can lead to more health decline (Jivraj et al. 2016). Participants’ observations of social disengagement due to health declines could explain why they continue to strive to be independent and help others who are unable to do things on their own. From these perspectives, this study reiterates the importance of social interaction in old age.

Findings also showed fewer opportunities for participants to meet others in their communities due to urbanization of services and social activities. Participants’ reliance on cars and difficulties accessing services in cities were also revealed. Fewer possibilities to meet people and fewer facilities nearby may put people at risk of becoming socially disengaged (Jivraj et al. 2016). Additionally, older adults living in rural areas were less likely to be socially engaged compared to older adults in urban areas (Vogelsang 2016). Therefore, even with transportation opportunities, difficulties to access city facilities can impede the social engagement of people from rural communities (Jivraj et al. 2016).

Digital technologies were perceived to enhance opportunities to contact others and access services. The importance of access to communication technologies to contact emergency care and receive support is consistent with findings in Reichstadt et al. (2007). That is, older adults felt security in knowing that they would receive care when their health declined. Besides feeling secure, participants had difficulties to imagine future participation needs while they were still well, which could be attributed to older adults viewing time as short and focusing on the present rather than the future (Carstensen et al. 2003). Moreover, though interest and curiosity were regarded as important for frequent use of digital technologies, digital technologies were acceptable as long as they provided satisfactory experiences and when support was available. Difficulties, such as receiving irrelevant information and having slower technologies, could impede an older person’s engagement. From a technology acceptance perspective (Chen and Chan 2014), ease of use and support from others are important factors in using digital technologies. From the socioemotional selectivity theory (Carstensen et al. 2003) perspective, older adults would disengage or not engage at all in activities involving unsatisfactory technology use. It is therefore relevant to consider using a co-creation or participatory approach in developing digital technologies to enhance relevance of content and usability, minimize problems, and adapt appropriate support.

### Methodological considerations

Older adults who are healthier and not in need of care are more likely to participate in research studies (Kammerer et al. 2019). If the study were to include older adults who experience health declines, recruitment would have been facilitated through healthcare channels rather than through communities and organizations. Within respective discussions, the moderators ensured that all participants had time to opine and share their views, as recommended by Dahlin-Ivanoff and Hultberg (2006) and Doody et al. (2013c), although participants who were more outspoken may have influenced how topics unfolded. All focus groups had both men and women represented, but gender perspectives were not specifically investigated. Though health information was not collected for ethical reasons, this choice could affect readers’ understanding of the context. Furthermore, the choice to collect data in rural communities in Northern Sweden highlighted particular social participation issues that older adults experienced, such as reduced mobility opportunities, increased geographical distances to social networks and essential community services, and changing demographics in rural areas. These issues put focus on the potential impact of digital technologies on older adults’ continued social participation and living at home.

Trustworthiness was strengthened by parallel coding, consensus discussions between all authors, and the authors belonging to different professions and perspectives. Though contexts are fluid and ever-changing, this study provided a glimpse into how older adults perceived their contexts. The time between data collection and reporting of results may have taken too long due to iterative analyses. However, the need to gain insight on relevant contexts in order to create and tailor opportunities supported by digital technologies and services for older adults is just as relevant now as it was during the initiation of this study.

## Conclusions

This study contributes to findings about contexts surrounding older adults’ social participation in a digital society. That is, social interaction is viewed as important in old age, but fewer opportunities for social interactions, in addition to fewer facilities and services in rural communities, can reduce social participation. Digital technologies are perceived to augment social opportunities and access to services, as well as provide a feeling of security, as long as they provide satisfactory experiences for the older adult. Thus, it is important to provide support when the decrease in social participation is undesired. Both co-creating usable digitalized services and facilitating satisfactory use of digital technologies could support older adults’ social participation through activities that they find relevant in their lives, and subsequently, might enable them to live longer at home.

## Footnotes

1 Quotations from the participants will be identified hereon with the focus group interview (number = focus group number, *a* or *b* = first or second interview) and participant gender (*M* = man, *W* = woman). For example, 2b W5 refers to a response in the focus group 2, second interview, by woman 5.

## References

[CR1] Aw S, Koh G, Oh Y, Wong M, Vrijhoef HM, Harding S, Hildon ZL (2017). Explaining the continuum of social participation among older adults in Singapore: from ‘closed doors’ to active ageing in multi-ethnic community settings. J Aging Stud.

[CR2] Barnes LL, de Leon CFM, Bienias JL, Evans DA (2004). A longitudinal study of black–white differences in social resources. J Gerontol Soc Sci.

[CR3] Bergström A (2017). Digital equality and the uptake of digital applications among seniors of different age. Nord Rev.

[CR4] Carstensen LL, Isaacowitz DM, Charles ST (1999). Taking time seriously: a theory of socioemotional selectivity. Am Psychol.

[CR5] Carstensen L, Fung H, Charles S (2003). Socioemotional selectivity theory and the regulation of emotion in the second half of life. Motiv Emot.

[CR6] Chen K, Chan AHS (2014). Gerontechnology acceptance by elderly Hong Kong Chinese: a senior technology acceptance model (STAM). Ergonomics.

[CR7] Conway F, Magai C, Jones S, Fiori K, Gillespie M (2013). A six-year follow-up study of social network changes among African-American, Caribbean, and U.S.-Born Caucasian urban older adults. Int J Aging Hum Dev.

[CR8] Dahlin-Ivanoff S, Hultberg J (2006). Understanding the multiple realities of everyday life: basic assumptions in focus group methodology. Scand J Occup Ther.

[CR9] Davidsson P, Thoresson A (2017) Svenskarna och internet 2017. https://www.iis.se/fakta/svenskarna-och-internet-2017/. Accessed 6 Apr 2018

[CR10] Davidsson P, Palm M, Mandre ÅM (2018) Svenskarna och internet 2018. https://2018.svenskarnaochinternet.se. Accessed 8 Oct 2018

[CR11] Digitaliseringskommissionen (2016) För digitalisering i tiden (SOU 2016:89). Elanders Sverige AB, Stockholm. http://www.regeringen.se/4af25c/contentassets/f7d07b214e2c459eb5757cea206e6701/sou-2016_89_webb.pdf. Accessed 30 Nov 2018

[CR12] Doody O, Slevin E, Taggart L (2013). Focus group interviews in nursing research: part 1. Brit J Nurs.

[CR13] Doody O, Slevin E, Taggart L (2013). Focus group interviews. Part 3: analysis. Brit J Nurs.

[CR14] Doody O, Slevin E, Taggart L (2013). Preparing for and conducting focus groups in nursing research: part 2. Brit J Nurs.

[CR15] Fischl C, Asaba E, Nilsson I (2017). Exploring potential in participation mediated by digital technology among older adults. J Occup Sci.

[CR16] Graneheim UH, Lundman B (2004). Qualitative content analysis in nursing research: concepts, procedures and measures to achieve trustworthiness. Nurse Educ Today.

[CR17] Graneheim UH, Lindgren B-M, Lundman B (2017). Methodological challenges in qualitative content analysis: a discussion paper. Nurse Educ Today.

[CR18] Ihm J, Hsieh YP (2015). The implications of information and communication technology use for the social well-being of older adults. Inf Commun Soc.

[CR19] Internetstiftelsen (2019) Svenskarna och internet 2019. https://svenskarnaochinternet.se/rapporter/svenskarna-och-internet-2019/. Accessed 4 Dec 2019

[CR20] Jivraj S, Nazroo J, Barnes M (2016). Short- and long-term determinants of social detachment in later life. Ageing Soc.

[CR21] Kammerer K, Falk K, Herzog A, Fuchs J (2019) How to reach ‘hard-to-reach’ older people for research: the TIBaR model of recruitment. Survey methods: insights from the field. https://surveyinsights.org/?p=11822. Accessed 24 Jun 2019

[CR22] Larsson E, Larsson-Lund M, Nilsson I (2013). Internet based activities (IBAs): seniors’ experiences of the conditions required for the performance of and the influence of these conditions on their own participation in society. Educ Gerontol.

[CR23] Leist AK (2013). Social media use of older adults: a mini-review. Gerontol Regen Technol Sect.

[CR24] Levasseur M, Richard L, Gauvin L, Raymond É (2010). Inventory and analysis of definitions of social participation found in the aging literature: proposed taxonomy of social activities. Soc Sci Med.

[CR25] Martinson M, Minkler M (2006). Civic engagement and older adults: a critical perspective. Gerontologist.

[CR26] Näsi M, Räsänen P, Sarpila O (2012). ICT activity in later life: internet use and leisure activities amongst senior citizens in Finland. Soc Behav Health Perspect.

[CR27] Olphert W, Damodaran L (2013). Older people and digital disengagement: a fourth digital divide?. Gerontology.

[CR28] Ragnedda M (2017). The third digital divide: a Weberian approach to digital inequalities.

[CR29] Regeringskansliet (2017) För ett hållbart digitaliserat Sverige—en digitaliseringsstrategi (N2017/03643/D). http://www.regeringen.se/informationsmaterial/2017/05/for-ett-hallbart-digitaliserat-sverige—en-digitaliseringsstrategi/. Accessed 30 Nov 2018

[CR30] Reichstadt J, Depp C, Palinkas L, Jeste D (2007). Building blocks of successful aging: a focus group study of older adults’ perceived contributors to successful aging. Am J Geriatr Psychiatry.

[CR31] Sandelowski M, Leeman J (2012). Writing usable qualitative health research findings. Qual Health Res.

[CR32] Schwartz E, Litwin H (2018). Social network changes among older Europeans: the role of gender. Eur J Ageing.

[CR33] Selwyn N (2006). Digital division or digital decision? A study of non-users and low-users of computers. Poetics.

[CR34] Skjutar A, Schult M-L, Christensson K, Müllersdorf M (2010). Indicators of need for occupational therapy in patients with chronic pain: occupational therapists’ focus groups. Occup Ther Int.

[CR35] Statistics Sweden (2012) Swedish standard classification of occupations. https://www.scb.se/en/finding-statistics/statistics-by-subject-area/other/other/other-publications-non-statistical/pong/publications/swedish-standard-classification-of-occupations-2012/. Accessed 16 Apr 2019

[CR36] Stewart DW, Shamdasani PN, Rook DW (2007). Focus groups: theory and practice.

[CR37] Twigg J (2007). Clothing, age and the body: a critical review. Ageing Soc.

[CR38] United Nations Educational, Scientific, and Cultural Organization (2012) International Standard Classification of Education ISCED 2011. UNESCO Institute for Statistics, Montreal, QC, Canada. http://uis.unesco.org/sites/default/files/documents/international-standard-classification-of-education-isced-2011-en.pdf. Accessed 16 Apr 2019

[CR39] Vogelsang E (2016). Older adult social participation and its relationship with health: rural–urban differences. Health Place.

[CR40] Winterton R, Clune S, Warburton J, Martin J (2014). Local governance responses to social inclusion for older rural Victorians: building resources, opportunities and capabilities. Australas J Ageing.

[CR41] World Health Assembly (2018) Seventy-first world health assembly—agenda item 12.4 digital health (WHA71.7). http://apps.who.int/gb/ebwha/pdf_files/WHA71/A71_R7-en.pdf?ua=1. Accessed 7 Jan 2019

[CR42] World Health Organization (2002) Towards a common language for functioning, disability and health: ICF. World Health Organization, Geneva. https://www.who.int/classifications/icf/icfbeginnersguide.pdf?ua=1. Accessed 24 Jun 2019

[CR43] World Health Organization (2015) World report on ageing and health. WHO Press, Geneva. http://apps.who.int/iris/bitstream/10665/186463/1/9789240694811_eng.pdf?ua=1. Accessed 21 Aug 2017

